# DAYSLEEPER: a nuclear and vesicular-localized protein that is expressed in proliferating tissues

**DOI:** 10.1186/1471-2229-13-211

**Published:** 2013-12-12

**Authors:** Marijn Knip, Steven Hiemstra, Afke Sietsma, Marina Castelein, Sylvia de Pater, Paul Hooykaas

**Affiliations:** 1 Department of Molecular and Developmental Genetics, Institute of Biology, Leiden University, Sylviusweg 72, Leiden 2333 BE, The Netherlands; 2 Current address: Department of Plant and Environmental Sciences, University of Copenhagen, Thorvaldsensvej 40, Frederiksberg C 1871, Denmark; 3 Current address: Department of Toxicology, Leiden/Amsterdam Center for Drug Research, Leiden University, Einsteinweg 55, Leiden 2333 CC, The Netherlands; 4 Current address: Department of Ecology and Physiology of Plants, Faculty of Earth and Life Sciences, Vrije Universiteit Amsterdam, de Boelelaan 1087, Amsterdam 1081 HV, The Netherlands

## Abstract

**Background:**

*DAYSLEEPER* is a domesticated transposase that is essential for development in *Arabidopsis thaliana* [Nature, 436:282–284, 2005]. It is derived from a hAT-superfamily transposon and contains many of the features found in the coding sequence of these elements [Nature, 436:282–284, 2005, *Genetics*, 158:949–957, 2001]. This work sheds light on the expression of this gene and localization of its product in protoplasts and *in planta*. Using deletion constructs, important domains in the protein were identified.

**Results:**

*DAYSLEEPER* is predominantly expressed in meristems, developing flowers and siliques. The protein is mainly localized in the nucleus, but can also be seen in discrete foci in the cytoplasm. Using several vesicular markers, we found that these foci belong to vesicular structures of the trans-golgi network, multivesicular bodies (MVB’s) and late endosomes. The central region as well as both the N- and the C-terminus are essential to DAYSLEEPER function, since versions of DAYSLEEPER deleted for these regions are not able to complement the *daysleeper* phenotype. Like hAT-transposases, we show that DAYSLEEPER has a functionally conserved dimerization domain [*J Biol Chem,* 282:7563–7575, 2007].

**Conclusions:**

DAYSLEEPER has retained the global structure of hAT transposases and it seems that most of these conserved features are essential to DAYSLEEPER’s cellular function. Although structurally similar, DAYSLEEPER seems to have broadened its range of action beyond the nucleus in comparison to transposases.

## Background

After being discovered by Barbara McClintock in the 1940’s, transposable elements (TE’s) were long viewed as integral constituents of the so-called “junk-DNA” [1]. These genomic regions were generally considered to represent non-coding, non-functional sequences. In the past ~20 years, however, the view of transposons has changed dramatically and they have made a comeback into the spotlights. TE’s are now thought to be the most important drivers of genome evolution, since they are thought to be responsible for a plethora of ways to influence genes, gene expression, and genome structure [2-4]. TE’s have contributed substantially to the protein coding capacity of their host genomes through the incorporation of transposon genes sequences into functional host genes [5]. In plants, a good example of molecular domestication of a transposase gene concerns the FAR1/FHY3 gene-family. This transcription factor gene family is evolutionary derived from the transposase gene of a MULE-type DNA transposon, but is now involved in the far-red light response [6]. DNA transposons code for transposases that can recognize and excise the entire element from the genome in a cut-paste fashion. It is assumed that genes in the FAR1/FHY3 family have evolved to encode proteins which use the DNA-binding capacity to control gene expression [6]. Many genes in various genomes have been uncovered over the years that are the result of molecular domestication of transposase genes [7]. *DAYSLEEPER* was described in 2005 as the first essential transposase-derived gene in Arabidopsis [8]. DAYSLEEPER structurally resembles a hAT transposase. DAYSLEEPER was identified by its ability to bind the promoter of the DNA-damage response gene Ku70 *in vitro* and is thought to influence transcription of other genes [8]*.* DAYSLEEPER harbors an arginine and lysine-rich nuclear localization signal (NLS), “KRRKKKK”, and was found to mainly be nuclear localized in Arabidopsis protoplasts. The NLS is followed by a BED-type zinc finger and 6 identifiable hAT blocks (A to F), but lacks the amino acids essential for mobility [8,9]. hAT Blocks D, E and F make up a hAT dimerization domain [10,11]. These hAT blocks are defining characteristics of hAT transposases in all species, although not all transposases possess all six blocks [10]. *DAYSLEEPER* is most likely derived from the Ac cluster elements within the hAT family [8,9]. *DAYSLEEPER*-like genes have been identified in various species, ranging from basal angiosperms to dicotyledonous species. These so-called *SLEEPER*-genes possess three conserved *SLEEPER*motifs, of which the third overlaps largely with hAT block E [9].

Here, we investigated the expression pattern of *DAYSLEEPER*, assessed functional complementation of the *daysleeper* phenotype with different deletions of the *DAYSLEEPER* coding sequence and studied its cellular localization *in planta* using fluorescent protein fusions.

## Results

### DAYSLEEPER expression

**Figure 1:**
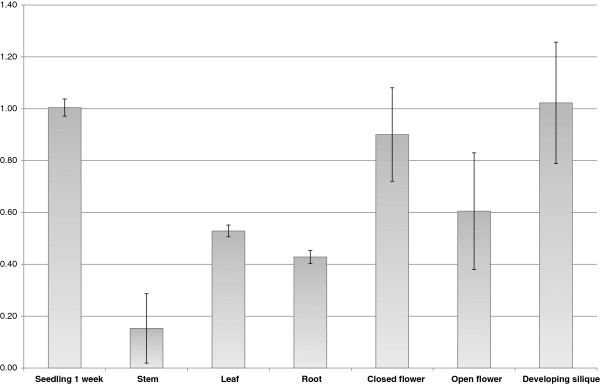
**qRT-PCR analysis of *DAYSLEEPER *expression in seedlings and various tissues of mature plants.** The relative expression of *DAYSLEEPER* in different organs of 4-week-old mature plants is set against the expression of *DAYSLEEPER* in whole seedlings. The experiments were performed *in triplo* and the error-bars reflect the variation between these experiments. Expression was normalized against *β-6-TUBULIN* expression.

**Figure 2:**
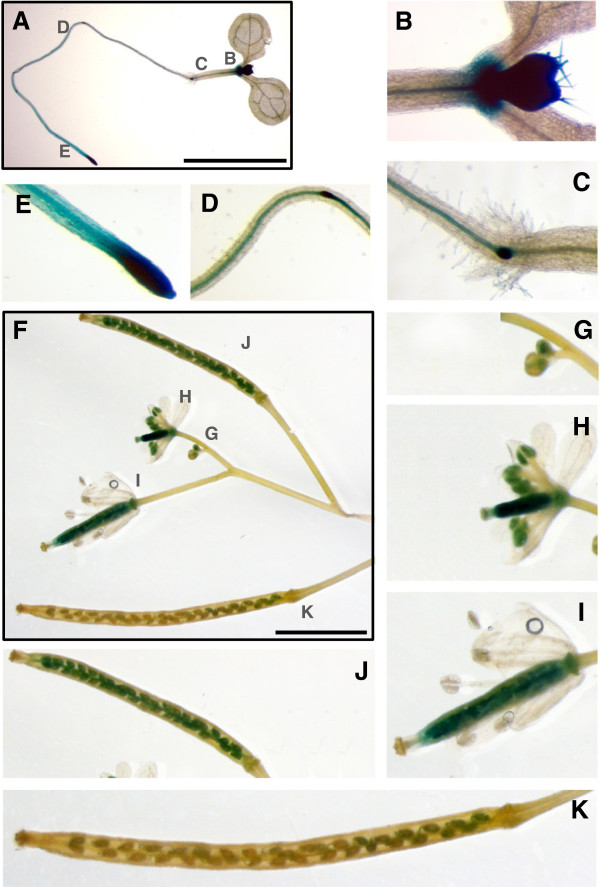
**GUS expression in seedlings, flowers buds, mature flowers and developing siliques. A** depicts a seedling, one week post germination, with enlarged areas shown in B-E. **B**: shoot apex and developing leaf. **C**: central stele, root crown. **D**: secondary stele with root meristem. **E**: root apex. **F**: depicts an inflorescence displaying several flowers and developing fruits in different stages, with enlarged areas shown in G-K. **G**: closed flower buds. **H**: flower with developing fruit. **I:** developing fruit. **J**: young silique. **K**: mature silique. The scale bars in **A** and **F** represent 5 mm.

To analyze the expression pattern of the *DAYSLEEPER* gene, qRT-PCR was performed to measure *DAYSLEEPER* transcript levels. *DAYSLEEPER* expression was found in all tissues analyzed. Expression levels were set against the expression levels found in material from one-week-old whole seedlings, using β -6-*TUBULIN* as a control (Figure 1). Relative expression in seedlings was 2 times higher as compared to leaf tissue of 4-week-old plants. Expression in stem tissue was low. Higher expression was found in flowers and developing siliques (Figure 1). To obtain a more detailed expression pattern, promoter-reporter constructs were created and studied *in planta*. Analysis of plant lines containing a 3.6 kb stretch of DNA directly upstream of the DAYSLEEPER start codon, including the 5′ UTR, fused to a *mGFP5:gusA* gene-construct (pSDM4328), showed that the *DAYSLEEPER* promoter was most active in the root apical meristem, secondary root meristems and the root central stele (Figure 2A-E). In the upper part of the seedling, expression was found in the shoot meristem and the embryonic cotyledons (Figure 2B). As the plant developed, expression was found mainly in proliferating tissues. Strong expression was found in the developing flower bud (Figure 2G). The developing pistil and the anthers displayed high expression levels as the flower developed (Figure 2H-I). In the anthers, expression diminished as the flower reached full maturation (Figure 2I). The expression in the pistil initially was rather uniform, but after fruit initiation was exclusively localized in developing seeds at later stages (Figure 2J-I). In mature siliques and seeds no expression activity was observed anymore (Figure 2K). No difference in the pattern of *gusA* expression was detected between plants containing the 1 kb or 3.6 kb *DAYSLEEPER* upstream sequence fragments, although expression levels in plants containing the 1 kb promoter fragment seemed higher overall (results not shown).

### DAYSLEEPER localization

#### Complementation of daysleeper with fluorescent protein fusion constructs

**Figure 3:**
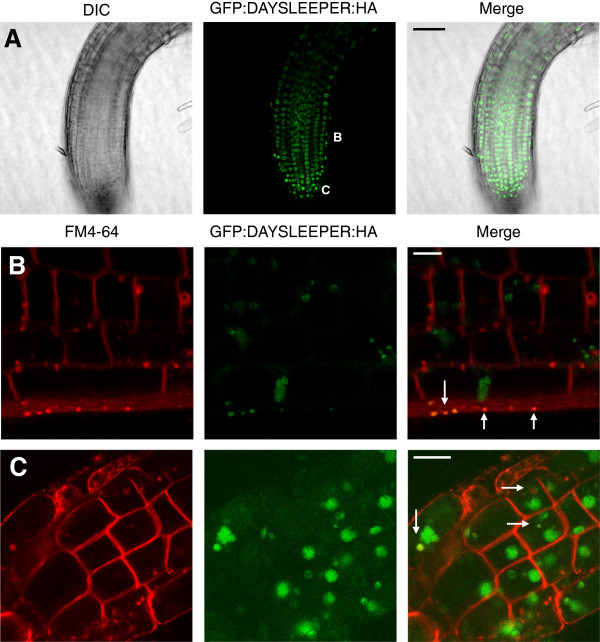
**Localization of fluorescent DAYSLEEPER fusions in Arabidopsis root cells. A**. An overview of the apical part of a seedling root, showing the predominant nuclear localization of GFP:DAYSLEEPER:HA. The white “B” and “C” indicate at which position of the root the pictures in panel **B** and **C** were taken (B and C are derived from different roots). **B**. depicts root epidermis and cortex cells and **C**. root cap cells, showing vesicles and membranes (FM4-64) or GFP fluorescence (DAYSLEEPER), or both (merge). Vesicular colocalizations are marked with arrows. The scale bar in **A** represents 50 μm. The scale bars in **B** and **C** represent 10 μm.

*GFP:DAYSLEEPER:HA* and *DAYSLEEPER:YFP:HA* harboring plants were created in a *DAYSLEEPER (daysleeper)* heterozygous background. These fusion proteins were expressed under control of the native *DAYSLEEPER* promoter, including the 5′ UTR. The *GFP:DAYSLEEPER:HA* construct was able to complement the *daysleeper* phenotype and fully restore the wild-type phenotype in the next generation, whereas the *daysleeper* phenotype could not be restored by the *DAYSLEEPER:YFP:HA* construct. Plant tissues were observed using confocal microscopy to study DAYSLEEPER localization *in planta.* GFP:DAYSLEEPER:HA was found in the nucleus of all cells of the plants (Figure 3A). Due to the depicted focal plane, Figure 3B and C do not show a nuclear signal for every cell, but nuclear signals were observed in all cells. In the elongation zone of the root and the root-tip, a cytoplasmic localization was also observed (Figure 3B and C) in both primary and secondary roots. These fluorescent signals were present in vesicle-like structures, which partly overlapped with the staining pattern of the membrane-specific fluorescent dye FM4-64 (Figure 3B-C) [12]. This was most pronounced in the epidermis of the root.

#### DAYSLEEPER localizes to multivesicular bodies, late endosomes and the trans-golgi network

**Figure 4:**
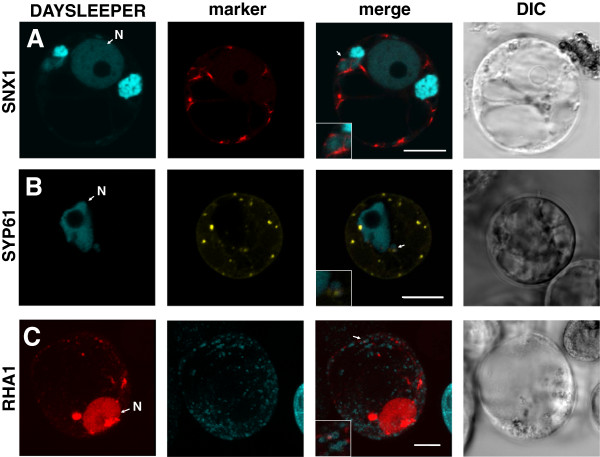
**Localization of fluorescent-tagged DAYSLEEPER in protoplasts.** Arabidopsis protoplasts transformed with **A**. *35S::Cerulean:DAYSLEEPER* and *35S::SNX1:mRFP1* constructs, **B**. *35S::Cerulean:DAYSLEEPER* and *35S::YFP:SYP61* constructs and **C**. 35S::*TagRFP:DAYSLEEPER* and *35S::RHA1:eCFP* constructs. From left to right, the panels depict fluorescent-tagged DAYSLEEPER (DAYSLEEPER), a fluorescent-tagged marker gene (marker), a composite image (merge) and a DIC image (DIC). The areas indicated by arrows are shown enlarged. The nucleus is designated with an “N”. The scale bars represent 10 μm.

N-terminal TagRFP- or Cerulean- tagged DAYSLEEPER fluorescent fusions were expressed under control of the strong CaMV 35S promoter in Arabidopsis protoplasts. We found that they localize in nuclei in all protoplasts, as expected from previous experiments [9], but also in multi-vesicular structures (Figure 4A) and smaller vesicular structures (Figure 4C) in 32% of protoplasts (SD = 3%, n = 3, 185 cells counted in total). To further investigate the observed vesicular localization of fluorescent DAYSLEEPER fusion proteins, we used fluorescent marker constructs for different vesicular organelles. Since we found that DAYSLEEPER interacts with a subunit of the ESCRTIII machinery (M. Knip, unpublished data), we suspected DAYSLEEPER localization to be in late endosomes and multi-vesicular bodies (MVBs). In order to verify this localization, co-expression was analyzed in protoplasts in which the *SNX1*, *SYP61* and *RHA1* genes were expressed tagged with a different fluorescent protein. SYP61 and SNX1 are both found in the trans-golgi network, but SNX1 localizes to sorting endosomes as well [13,14]. RHA1 localizes to late endosomes and MVB’s [15]. DAYSLEEPER-containing multivesicular structures are sometimes also positive for SNX1, which appears to be on the outer membrane of the multivesicular structure, while the DAYSLEEPER fusion protein seems to be internalized (Figure 4A). For SYP61 and RHA1 colocalization with DAYSLEEPER-fusion constructs we observed similar patterns. We found vesicles that contained two fluorescent constructs, (Figure 4B and C) but the majority of vesicles were positive only for either the DAYSLEEPER-fusion protein or the marker gene. We propose that DAYSLEEPER-fusion proteins accumulate in larger vesicular structures, which are most likely MVBs (Figure 4).

### DAYSLEEPER structure

#### DAYSLEEPER can homodimerize through its hAT-dimerization domain

**Figure 5:**
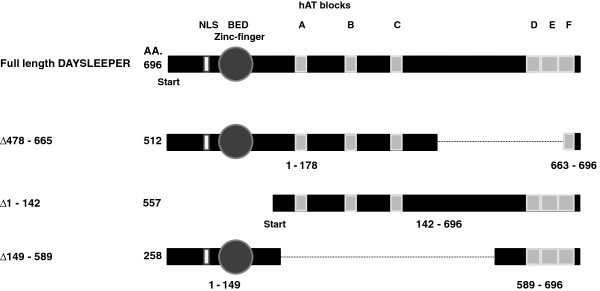
**DAYSLEEPER structure and deletions.** DAYSLEEPER is 696 amino acids long and has a NLS, a BED zinc finger and hAT blocks. The hAT blocks are located at the following positions: Block **A**: 231–243, **B**: 333–345, **C**: 397–406, **D**: 607–617, **E**: 621–645, **F**: 647–674. Three shortened coding sequences are displayed, which are deleted at either the N-terminus (Δ1-142), the central part (Δ149-589), or the C-terminus (Δ478-665). Sizes of the coding sequences of different constructs are shown, as well as the position of deletions.

**Figure 6:**
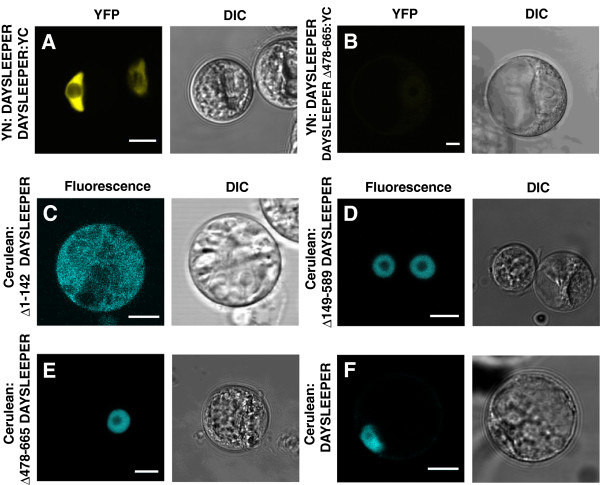
**DAYSLEEPER dimerization and localization of deletion constructs in protoplasts. A**. Bimolecular fluorescence complementation of YN:DAYSLEEPER and DAYSLEEPER:YC. **B**. Bimolecular fluorescence complementation of YN:DAYSLEEPER and Δ478-665 DAYSLEEPER:YC. **C**. Cerulean: Δ1-142 DAYSLEEPER. **D**. Cerulean: Δ149-589 DAYSLEEPER **E**. Cerulean: Δ478-665 DAYSLEEPER. **F**. Cerulean:DAYSLEEPER. Left panels: fluorescent-tagged DAYSLEEPER. Right panels: DIC images. The scale bars represent 10 μm.

Six conserved motifs of amino acid sequences, so-called hAT-blocks, have been found that are characteristic for hAT transposases in different species (Figure 5) [16]. Ac-elements do not always possess all hAT-blocks; about 50% contain blocks A, B and/or C and about 75% contains blocks D, E and/or F [10]. *DAYSLEEPER* contains signatures of all blocks, although hAT block B is somewhat different from the reported consensus [10]. In DAYSLEEPER, this 13 amino acid (AA) sequence lacks a histidine at position 7, as well as the asparagine and leucine at position 10 and 11 [10]. The highly conserved tryptophan at position 26 is replaced by a leucine in DAYSLEEPER. Block F (28 AA’s) is clearly identifiable, but the first 6 AA’s of this sequence are different from the consensus sequence. Of these six blocks, named A to F, hAT block D, E and F make up a dimerization domain in transposases. Since these blocks are largely conserved in DAYSLEEPER, we tested if DAYSLEEPER is able to dimerize via Bimolecular Fluorescence Complementation (BiFC) [17]. We created an N-terminal fusion of DAYSLEEPER fused to the N-terminal part of YFP (AA 1–155, YN) and a C-terminal fusion of DAYSLEEPER fused to the C-terminal half of YFP (AA 155–238, YC) (Figure 6) [18]. Co-transformation of these constructs (*YN:DAYSLEEPER and DAYSLEEPER:YC*), both controlled by the strong CaMV 35S promoter, resulted in fluorescence reconstitution of YFP in Arabidopsis protoplasts (Figure 6A). A fluorescent signal was exclusively found in the nucleus. We also tested interaction of *YC:DAYSLEEPER* and *YN:DAYSLEEPER.* In this case localization was observed in the nucleus, but also in vesicular structures (results not shown). However this combination produced a signal only in a few non typical-cells, irrespective of the transformation efficiency of protoplasts. Constructs did not display fluorescence on their own, or in combination with a non-fused split-YFP moiety. This shows that DAYSLEEPER is able to dimerize. To test whether the hAT-blocks D, E and F were indeed responsible for dimerization, we co-transformed protoplasts with a *YN:DAYSLEEPER* construct and a *Δ478-665 DAYSLEEPER:YC* construct. The shortened version of DAYSLEEPER lacks a large part of its C-terminus (Figure 6B). Although some background fluorescence can be observed, we were not able to find a reconstituted YFP signal in these experiments (Figure 5B), indicating that the C-terminal hAT blocks D,E and F are indeed responsible for homodimerization.

#### Full-length DAYSLEEPER coding sequence is required for complemention of the daysleeper phenotype

In order to study the biological function of different regions of the *DAYSLEEPER* coding sequence *in vivo,* complementation was attempted of *daysleeper* mutant plants with 3 different deletion versions of the *DAYSLEEPER* coding sequence (Figure 6). All shortened coding sequences (Figure 6) were preceded by the native upstream sequence of *DAYSLEEPER*, including the 5′ UTR. Plants heterozygous for an insertion in *DAYSLEEPER* were transformed with pEARLEYGATE301 vectors with *pDAYSLEEPER::Δ1-142 DAYSLEEPER:HA*, *pDAYSLEEPER::Δ149-589 DAYSLEEPER:HA* or *pDAYSLEEPER::Δ478-665 DAYSLEEPER:HA* constructs (Additional file 1: Table S3). Progeny (T1) was grown on double selection: sulfadiazine for selection of the T-DNA insert in the *DAYSLEEPER* gene and PPT for selection of the construct with the shortened *DAYSLEEPER* gene. Presence of the complementing construct in each line was confirmed by PCR on genomic DNA. Resistant seedlings were grown to maturity and progeny of these plants (T2) were grown on double selection again. Amongst the T2 progeny grown on double selection were in all cases (for each construct 8 or 9 independent lines), still plantlets with the *daysleeper* phenotype (Additional file 2: Data Sheet S1). Therefore, we have to conclude that none of the three constructs can complement for the absence of an intact *DAYSLEEPER* gene. This indicates that all three regions deleted in the three partial DAYSLEEPER versions, the N-terminus, the central part and the C-terminus are essential for its function.

#### DAYSLEEPER’s N-terminus is essential for nuclear localization

Full-length and shortened versions of DAYSLEEPER (Figure 5) were fused to the fluorophore Cerulean (pSDM4367-4369) and were transformed into protoplasts (Figure 6C-F). Localization of DAYSLEEPER with central (Δ149-589) and C-terminal (Δ478-665) deletions were similar to the localization of fluorescent fusions of full-length DAYSLEEPER (Figure 6DEF), whereas the N-terminal truncated construct (Δ1-142) never showed a nuclear signal and was uniformly localized in the cytosol (Figure 6C). The lack of a nuclear signal is in line with the location of the proposed NLS, discussed in the first paragraph, which is missing in the Δ1-142 deleted protein.

## Discussion

We found that *DAYSLEEPER* is predominantly expressed in proliferating tissues (Figures 1 and 2). This is in concordance with the important role that *DAYSLEEPER* has in development and the retarded growth and flower phenotype of plants overexpressing *DAYSLEEPER*[1]. We used 3.6 kb of sequence immediately preceding the start-codon of the DAYSLEEPER gene to drive the expression of the *gusA*-gene (Figure 2). We also used shorter (1 kb) and longer (6 kb) stretches, but these results were comparable to the data shown in Figure 2 (data not shown).

DAYSLEEPER does not seem to possess any known protein domains other than those also found in hAT transposases. Although DAYSLEEPER thus seems very similar to canonical hAT transposases, it must be noted that hAT blocks are rather loosely defined. DAYSLEEPER contains the signatures of these blocks, but few conclusions about function can be drawn from this, since the blocks are defined on the basis of homology and not functionality [10]. However, blocks D, E and F have been found to be necessary for dimerization, which makes it likely that DAYSLEEPER would also be able to form dimers. We have shown in this manuscript in BiFC experiments that DAYSLEEPER indeed forms homodimers (Figure 6).

The localization of DAYSLEEPER was studied by fusion of the protein to fluorescent proteins. It appeared that the localization of DAYSLEEPER partially depended on whether the fluorescent moiety was fused to its C- or N-terminus. When the fluorescent moiety was fused to the C-terminus of DAYSLEEPER, an exclusive nuclear localization was observed in contrast to N-terminally tagged DAYSLEEPER fusion-proteins, which showed both a nuclear and a vesicular localization. The N-terminal part containing a putative nuclear localization sequence turned out to be responsible for the nuclear localization as tagged constructs lacking this part of DAYSLEEPER showed a cytoplasmic localization. Although we did find a few cells expressing the N-terminal fluorescent-tagged Δ1-142 DAYSLEEPER fusion-protein, the majority of cells died after transformation indicative of the toxicity of this particular fusion protein. The C-terminally fluorescent-tagged Δ1-142 DAYSLEEPER fusion-protein did not display this toxicity. Although this protein showed a cytoplasmic localization, it was not localized in vesicular structures, (data not shown). This might hint to a role of a free C-terminus of the protein in vesicular localization. Dimerization of DAYSLEEPER was observed in nuclei of protoplasts. When using two N-terminal-tagged constructs also a vesicular localization was seen, again indicating the importance of a free C-terminus for the vesicular localization. However, there was no difference detectable in localization between N- or C-terminal-tagged fusion proteins *in planta.* These constructs seemed to be localized similarly; in the nucleus and in vesicular structures. C-terminal tagged DAYSLEEPER appeared non-functional, since it did not complement the *daysleeper* phenotype. We presume that the difference in localization found in protoplasts are due to the fact that the studied proteins were expressed at a much higher level than is the case *in planta*, where *DAYSLEEPER*-constructs were expressed under control of the native *DAYSLEEPER*-promoter.

A partially extranuclear localization was unexpected in light of DAYSLEEPER’s structure and suspected functionality. The protein was shown to bind to DNA suggesting that DAYSLEEPER plays a role in the nucleus, as was found for most domesticated transposases [7,8]. We speculate therefore that DAYSLEEPER is to some extent transported out of the nucleus by an interaction partner. We have found that DAYSLEEPER can bind Arabidopsis homologs of the ESCRTIII machinery subunit VPS2 (M. Knip, unpublished data). The ESCRTIII machinery is highly conserved and its main function is to snare off vesicles from membranes. We speculate that DAYSLEEPER’s binding to VPS2-homologs in Arabidopsis might facilitate the translocation of DAYSLEEPER from the nucleus to vesicles. VPS2.2 has recently been shown to be partially localized to nuclei in Arabidopsis roots [19]. Further studies will have to reveal the functional implications of this interaction. Based on our co-localization experiments (Figure 4), we speculate that DAYSLEEPER is transported from the nucleus through the trans-golgi network and targeted to MVB’s and late endosomes. We were not able to definitively show colocalization of MVB’s with DAYSLEEPER using our marker gene set, although in Figure 4A multi-vesicular structures can be seen, that are not stained by SNX1 and therefore might be MVB’s. Future analysis of localization should discern the precise nature of DAYSLEEPER localization, by analyzing constructs *in planta*, instead of in a semi-artificial protoplast system.

## Conclusions

DAYSLEEPER is a predominantly nuclear protein that is expressed mainly in meristems, developing flowers and fruits. It is able to dimerize, most likely enabled by its hAT transposase-like dimerization domain. Although nuclear in most cells, vesicular localization was observed in root-tips and in protoplasts. We hypothesize that the N-terminal “KRRKKKK” nuclear localization motif of DAYSLEEPER is responsible for its nuclear localization, and that interaction with other factors allows it to be present outside the nucleus. We propose DAYSLEEPER’s vesicular localization is situated in the trans-golgi network, late endosomes and MVBs.

## Methods

### Cloning

PCR primers and vectors can be found in Additional file 1: Table S3 and Additional file 3: Table S2, respectively. PCR reactions were performed using the Phusion® polymerase (Finnzymes®) with HF-buffer and recommended conditions, unless stated otherwise. Cloned PCR products were sequenced. Restriction enzymes were obtained from Fermentas®, except for AlwNI, AvaI and HpaI which were obtained from New England Biolabs (NEB®).

#### cDNA isolation

A λ-phage cDNA library constructed from auxin-treated Arabidopsis roots [20] was used as a template for the amplification of the full length coding sequences of *DAYSLEEPER*, *RHA1* and *SNX1 YFP:SYP61* was obtained from Prof. P. Pimpl [21].

#### Binary vectors for promoter analysis using the gusA reporter gene

*DAYSLEEPER* promoter constructs were made in pCAMBIA1304 (CAMBIA foundation) to obtain promoter-reporter gene fusions. First, to separate the *DAYSLEEPER* promoter from promoter elements present on the pCAMBIA1304 vector, λ phage HindIII DNA marker (New England Biolabs®) was digested using KpnI and BamHI and the 5 kb fragment was cloned into the respective sites in the multiple cloning sites (MCS) of pCAMBIA1304, resulting in pCAMBIA1304λ. Using primer combination MK3 and MK4, 6.1 kb of the *DAYSLEEPER* upstream region was amplified from genomic DNA. This fragment was subsequently cloned into pJET1.2 (Fermentas®), giving rise to pJET1.2 6.1 kb p*DAYSLEEPER*. Using NcoI and EcoRI, the 35S promoter of the pCAMBIA1304λ vector was replaced by 3.6 kb of the *DAYSLEEPER* promoter, resulting in pSDM4328. The same was done using the enzymes NcoI and XbaI, giving rise to a pCAMBIA1304λ vector with 1 kb of *DAYSLEEPER* upstream sequence cloned in its MCS, resulting in pSDM4327. In both plasmids (pSDM4327 and pSDM4328) the *mGFP5:gusA* coding sequence is preceded by *DAYSLEEPER* upstream sequence, which in turn is spaced from elements present in the pCAMBIA backbone by a 5 kb stretch of λ DNA.

#### Binary vectors for DAYSLEEPER complementation and YFP fusions

*DAYSLEEPER* coding sequence was amplified using RedTAQ (Sigma Aldrich®) and primers PB1 and PB2 and cloned into pGEMtEASY (Promega®) to give rise to pGEMtEASY::At3g42170 (pSDM2099) and cloned into pAS2-1 (CLONTECH) to give rise to pSDM2304. In order to delete the central region of *DAYSLEEPER*, pSDM2304 was digested with Age1 and AlwNI (New England Bioscience; NEB®), blunted with T4 polymerase (Fermentas®) and ligated, giving rise to a shortened *DAYSLEEPER* coding sequence (Δ149-589) (pSDM4415).

To create a C-terminal deletion (Δ478-665), pSDM2099 was digested with the restriction enzyme AvaI and subsequently ligated, deleting the sequence between the 2 AvaI sites. The coding sequence was subsequently obtained with NcoI and SmaI and cloned into pAS2-1 (CLONTECH®), resulting in pSDM4416. To join the Δ149-589 and Δ478-665 shortened coding sequences with the native *DAYSLEEPER* promoter, the pAS2-1 vectors containing the C-terminal and central truncated *DAYSLEEPER* coding sequence were cut using NcoI and HpaI. The 3.6 kb fragment directly upstream of the ATG of the *DAYSLEEPER* coding sequence was inserted, after having been isolated using the same restriction enzymes. This 3.6 kb fragment was obtained from the *DAYSLEEPER* upstream fragment described in the paragraph “*Binary vectors for promoter analysis using the gusA reporter gene”.*

To create an N-terminal deletion of the *DAYSLEEPER* coding sequence (Δ1-142), PCR primers MK39.1 and MK40 were used to amplify bases coding for amino acid 142 until the stop codon, adding an NcoI restriction site at the 5′end of the fragment and an EcoRI site flanking the stop codon at the 3′end. The resulting PCR fragment was cut with NcoI and EcoRI (NEB®) and used for cloning into the pJET1.2 6.1 kb p*DAYSLEEPER* vector described in the paragraph “*Binary vectors for promoter analysis using the gusA reporter gene”.* This plasmid was cut with NcoI and SmaI (NEB®) and ligated with the digested PCR fragment, to give rise to a vector with *DAYSLEEPER* upstream sequence directly fused to the N-terminal (Δ1-142) truncated coding sequence of *DAYSLEEPER*.

#### Gateway cloning of binary vectors

Using Gateway-compatible primers the promoter::coding sequence fusions described above were amplified. This was performed using a slightly modified standard PCR protocol using Phusion polymerase and HF-buffer (Finnzymes®). The annealing temperature was set at 65°C for all reactions. The primers used to amplify the different fragments can be found in Additional file 3: Table S2. PCR reactions were performed on ~0.5 ng plasmid template per reaction, except for the amplification of the native *DAYSLEEPER* upstream region and coding sequence, which were amplified directly from genomic DNA. All PCR fragments were subsequently cloned into the vector pDONR207 (Invitrogen®), using BP Clonase II (Invitrogen®). The resulting pENTR clones were recombined with a binary destination vector. The three *DAYSLEEPER* versions, Δ1-142, Δ149-589 and Δ478-665 with *DAYSLEEPER* upstream sequence were recombined into pEARLEYGATE301 [22], using LR Clonase II (Invitrogen®), giving rise to pSDM4323 to 4325. The full length genomic sequence of the *DAYSLEEPER* locus was recombined into pGREEN179YFP:HA [23] following the same method, giving rise to pSDM4322.

#### Gateway cloning of protoplast vectors

The pENTR clones described above were cloned into a pART7-derived plasmid containing the appropriate Gateway cassette in frame with a fluorophore coding region [24,25], using LR Clonase II (Invitrogen®), resulting in pSDM4337 and pSDM4341. For N-terminal fusions to fluorophores, HindIII fragments of the pSITEII 2C1 and 6C1 vectors [26] containing the expression cassettes were cloned into HindIII digested pSY vectors [18,27]. This gave rise to the pSYSAT6 2xp35S TagRFP Gateway and pSYSAT6 2xp35S Cerulean Gateway vectors, respectively (pSDM4366 and pSDM4376).

#### Cloning of the DAYSLEEPER coding sequence into the pSY vectors

For the Bimolecular fluorescence-complementation assay in Arabidopsis protoplasts, the DAYSLEEPER coding sequence was isolated and restriction sites added (see: Additional file 3: Table S2) using PCR, cloned into pJET1.2 (Fermentas®) and sequenced. The DAYSLEEPER coding sequence was isolated from pJET1.2 using the appropriate restriction enzymes and subsequently cloned into the pSY728, 735, 736 and 738 vectors [18] (Additional file 1: Table S3 and Additional file 3: Table S2).

### qRT-PCR Analysis

Seedlings were grown *in vitro* for 2 weeks, after being transferred to soil. Plants were grown with 12 hours of light at 21°C. Samples of *Arabidopsis thaliana* Col-0 were collected, flash-frozen in liquid nitrogen and stored at −80°C. The tissue was ground under liquid nitrogen in a TissueLyser II apparatus (Qiagen®). RNA was isolated with the RNeasy Mini Kit (Qiagen®) and 1 μg was treated with DNase I (Ambion®), according to the recommended protocol, with the addition of 0.5 μL RNasin (Promega®) per reaction. From each sample, 0.5 μg was used for subsequent random-primed cDNA synthesis, using an iScript cDNA kit (BioRad®). qRT-PCR was performed on 1 μl 20x diluted cDNA, using a standard PCR reaction mix for Phusion DNA polymerase (Finnzymes), with the addition of 1.25 μL 500x diluted SYBR Green (BioRad®) in DMSO. To measure *DAYSLEEPER* transcript levels, primer combination MK1/MK2 was used. Transcript levels were normalized against expression of the housekeeping gene β*-6-TUBULIN* (At5g12250). Primers were adopted from Czechowski *et al.*[28] (Additional file 1: Table S3). Experiments were performed in triplicates on a Chromo4 Real-Time PCR Detection system (Biorad®). Data were processed using the Opticon Monitor 3.1 software (Biorad®) and the GeNorm normalization procedure [29].

### Histochemical staining of gusA expressing plants

Seedlings of 10 days old were grown on solid ½ MS medium [30] and stained with bromo-4-chloro-3indolyl-Beta-D-glucuronic acid (X-GLUC). Organs (eg. flower buds, leaves, siliques) of mature plants grown on soil were cut off and stained with X-GLUC staining. Seedlings and various tissues were fixed in 90% acetone for 1 hour at −20°C, washed three times in 10 mM EDTA, 100 mM sodium phosphate (pH7.0), 2 mM K_3_Fe(CN)_6_ and subsequently stained for 2 h in 10 mM EDTA, 100 mM sodium phosphate (pH7.0), 1 mM K_3_Fe(CN)_6_, 1 mM K_4_Fe(CN)_6_ containing X-GLUC (Duchefa®). Tissue was post-fixed in ethanol-acetate (3:1), cleared in 70% ethanol and stored in 100 mM sodium phosphate (pH7.0).

### Arabidopsis plant, protoplast transformation and microscopic analysis

*Arabidopsis thaliana* ecotype *Columbia-0 (Col-0)* was used for floral dip transformation according to Clough and Bent, 1998 [31]*. Arabidopsis thaliana* (*Col-0)* suspension cells were used to isolate and transform protoplasts [32]. Protoplasts were observed after 16–18 hours of incubation at 25°C in the dark on a Zeiss Observer (Zeiss®) confocal microscope.

### Observation of fluorescent constructs in Arabidopsis tissues and protoplasts

Seedlings were taken directly from ½ MS solid plates and observed on a Zeiss Imager confocal microscope (Zeiss®) prepared on a glass slide with cover slip [31]. Older plants were dissected using a razor blade to allow observation of tissues using a glass slide and cover slip.

Fluorescent signals were visualized using a 63x oil objective on the Zeiss Imager and a 63x water objective with the Zeiss Observer confocal microscope. An Argon laser at 514 nm for excitation and a 530/600 nm band pass emission filter was used for GFP and YFP signals. FM4-64 was also excited with the 514 nm Argon laser and the emission was collected using a 530/600 nm band-pass filter. Cerulean was excited using a 458 nm laser and the emission was collected using a 475/525 nm band pass filter. TagRFP was visualized using a 543 nm laser and a 560/615 nm band pass filter. Images were processed using ImageJ [33] and Adobe Photoshop CS5 (Adobe®).

## Competing interests

The authors declare that they have no competing interests.

## Authors’ contributions

MK performed most of the experiments and data processing in this study. SdP contributed technical advice. MC performed the analysis of the promoter::*gusA* experiment. AS and MK performed experiments for the analysis of DAYSLEEPER dimerization in protoplasts. SH performed most of the protoplast work, including cloning, transformations and microscopy. MK, SdP and PJJH contributed to the study design and writing of the manuscript. All authors have read and approved the final manuscript.

## Supplementary Material

Additional file 1: Table S3 Plasmids used for localization of SLEEPER fusion proteins in protoplasts and complementation of the *daysleeper* phenotype in *Arabidopsis thaliana.* Collection number, brief description and purpose in this work are shown. Click here for file

Additional file 2 **Data Sheet S1.** Overview of the method of screening for complementation of the *daysleeper* phenotype with different shortened versions of *DAYSLEEPER.* Click here for file

Additional file 3: Table S2 PCR Primers. Primer names, descriptions and sequences are shown. Click here for file
