# Supplementary material for: DAYSLEEPER: a nuclear and vesicular-localized protein that is expressed in proliferating tissues
Source: BMC Plant Biol. 2013 Dec 12;13:211. doi: 10.1186/1471-2229-13-211 (PMC4029315; doi:10.1186/1471-2229-13-211)
Supplement: Additional file 1: Table S3 — Plasmids used for localization of SLEEPER fusion proteins in protoplasts and complementation of the daysleeper phenotype in Arabidopsis thaliana. Collection number, brief description and purpose in this work are shown. [file 1471-2229-13-211-S1.docx]

| **Collection number** | **Description** | **Purpose** |
| --- | --- | --- |
| pSDM2099 | pGEM-t Easy At3g42170 |  |
| pSDM4337 | pART7 p35S Gateway FLAG mRFP1 SNX1 | Addition of a fluorescent tag on the C-terminus of a gene for protoplast transformation |
| pSDM4341 | pART7 p35S Gateway FLAG eCFP RHA1 |  |
| pSDM4366 | pSYSAT6 2xp35S Cerulean Gateway DAYSLEEPER | Addition of a fluorescent tag on the N-terminus of a gene for protoplast transformation |
| pSDM4367 | pSYSAT6 2xp35S Cerulean Gateway DAYSLEEPER Δ1-142 |  |
| pSDM4368 | pSYSAT6 2xp35S Cerulean Gateway DAYSLEEPER Δ478-665 |  |
| pSDM4369 | pSYSAT6 2xp35S Cerulean Gateway DAYSLEEPER Δ149-589 |  |
| pSDM4376 | pSYSAT6 2xp35S TagRFP Gateway DAYSLEEPER |  |
| pSDM4384 | pSY 728 35S::EE:YN:DAYSLEEPER | Protoplast transformation vectors for BiFC assay |
| pSDM4385 | pSY 735 35S::DAYSLEEPER:YC:HA |  |
| pSDM4386 | pSY 736 35S::DAYSLEEPER:YN:EE |  |
| pSDM4387 | pSY 738 35S::HA:YC:DAYSLEEPER |  |
| pSDM4322 | pGREEN179 Gateway YFP:HA pDAYSLEEPER::DAYSLEEPER | Plant transformation vectors for complementation assays |
| pSDM4323 | pEARLEYGATE301 pDAYSLEEPER::DAYSLEEPER N-term. Truncation :HA |  |
| pSDM4324 | pEARLEYGATE301 pDAYSLEEPER::DAYSLEEPER Central. Truncation :HA |  |
| pSDM4325 | pEARLEYGATE301 pDAYSLEEPER::DAYSLEEPER C-term. Truncation :HA |  |
| pSDM4326 | pEARLEYGATE301 pDAYSLEEPER::GFP:DAYSLEEPER:HA |  |
| pSDM4327 | pCAMBIA1304 Lambda 1kb pDAYSLEEPER::mGFP5:GUSa | Plant transformation vectors for promoter activity assay |
| pSDM4328 | pCAMBIA1304 Lambda 3.6kb pDAYSLEEPER::mGFP5:GUSa |  |
| pSDM2304 | pAS2.1 At3g42170 (*DAYSLEEPER*) |  |
| pSDM4415 | pAS2.1 DAYSLEEPER Δ149-589 |  |
| pSDM4416 | pAS2.1 DAYSLEEPER Δ478-665 |  |
