# Supplementary material for: DAYSLEEPER: a nuclear and vesicular-localized protein that is expressed in proliferating tissues
Source: BMC Plant Biol. 2013 Dec 12;13:211. doi: 10.1186/1471-2229-13-211 (PMC4029315; doi:10.1186/1471-2229-13-211)
Supplement: Additional file 2 — Data Sheet S1. Overview of the method of screening for complementation of the daysleeper phenotype with different shortened versions of DAYSLEEPER. [file 1471-2229-13-211-S2.docx]

**Complementation of the *daysleeper* phenotype with shortened versions of the *DAYSLEEPER* coding sequence.**

Method of screening for wild-type *DAYSLEEPER* expression by rt-PCR.


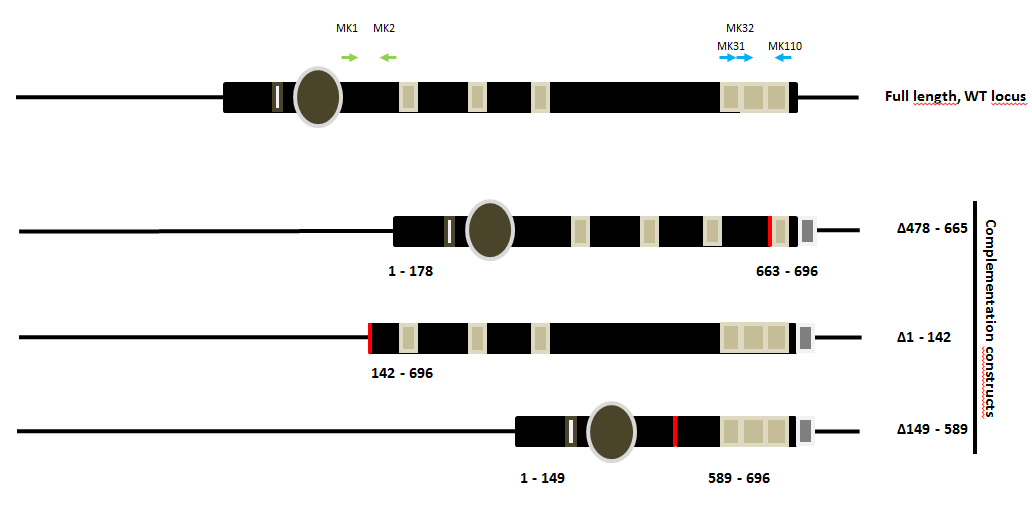
Above, the wild-type *DAYSLEEPER* locus is depicted and the three constructs we used in our complementation assay (Figure 5). The HA-tag is designated with a box on the C-terminal end of the complementation constructs. The red vertical lines indicate the position where the original coding sequence was deleted. At the top, primer binding site and primer names are depicted. The MK1-MK2 primer combination was used to screen for the absence of full-length *DAYSLEEPER* expression in plants transformed with N-terminally deleted and central region-deleted constructs, whereas MK31/32-MK110 were used in combination with the plants bearing C-terminally deleted constructs. These primer combinations do not produce a pcr-product in the respective shortened coding regions, but do produce a product when the full length *DAYSLEEPER* gene is being transcribed in a PCR reaction on cDNA.

Expression of the wild-type *DAYSLEEPER* gene was found in all samples tested. Primers MK1 and 2 were also used for the rt-PCR analysis in this work (Primer list S2). The other primers are listed below.

| Primer Name | Sequence |
| --- | --- |
| MK31 | ACGACATCTGAAGGTGGGAA |
| MK32 | AGCTTGTCGAGTTCAGTTTC |
| MK110 | CTTCAGATTTGATGGTAGCAC |

Below is an example of a *DAYSLEEPER^-/-^ pDAYSLEEPER::Δ149-589 DAYSLEEPER:HA* seedling. The plant doesn’t develop further than this stage and displays the typical *daysleeper* phenotype (1).


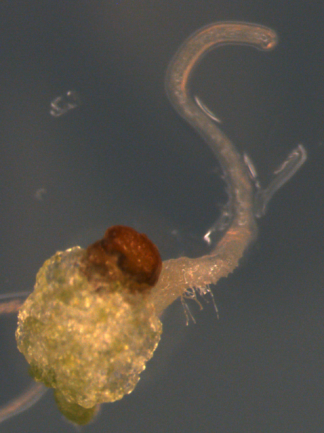


***DAYSLEEPER^-/-^***

***pDAYSLEEPER::Δ149-589 DAYSLEEPER:HA***
