# Supplementary material for: DAYSLEEPER: a nuclear and vesicular-localized protein that is expressed in proliferating tissues
Source: BMC Plant Biol. 2013 Dec 12;13:211. doi: 10.1186/1471-2229-13-211 (PMC4029315; doi:10.1186/1471-2229-13-211)
Supplement: Additional file 3: Table S2 — PCR Primers. Primer names, descriptions and sequences are shown. [file 1471-2229-13-211-S3.docx]

| **Primer** | **Description** | **Sequence** |
| --- | --- | --- |
| AS01 | DAYSLEEPER CDS cloning into pSY735/36, forward primer Sal1 | CAGTCGACTATGGAAGTGTACAATGACGATAC |
| AS02 | DAYSLEEPER CDS cloning into pSY735/36, reverse primer Spe1 | CTACTAGTCTATGCTTCAGATTTGATGGTAG |
| AS03 | DAYSLEEPER CDS cloning into pSY728/38, forward primer Nco1 | CGCGCCATGGATATGGAAGTGTACAATGACGATAC |
| AS04 | DAYSLEEPER CDS cloning into pSY728/38, reverse primer Not1 | GCGCGGCCGCTGTGCTTCAGATTTGATGGTAGCAC |
| B6Tf | Beta-6-Tubulin qRT-PCR forward | ACCACTCCTAGCTTTGGTGATCTG |
| B6Tr | Beta-6-Tubulin qRT-PCR reverse | AGGTTCACTGCGAGCTTCCTCA |
| MK01 | DAYSLEEPER qRT-PCR forward | ACAATGACAACAACCCACTG |
| MK02 | DAYSLEEPER qRT-PCR reverse | CACGAACGAGACAAAACCTG |
| MK03 | DAYSLEEPER promotor amplification forward | CCATGGTCTTTGCAACATAACATAAAAAGG |
| MK04 | DAYSLEEPER promotor amplification reverse | GTGATGGCATAGCATATTG |
| MK147 | SNX1 forward primer, gateway | GGGGACAAGTTTGTACAAAAAAGCAGGCTCCATGGAGAGCACGGAGCAGCCGAGG |
| MK148 | SNX1 reverse primers, gateway | GGGGACCACTTTGTACAAGAAAGCTGGGTGGACAGAATAAGAAGCTTCAAGTTTG |
| MK151 | DAYSLEEPER reverse primer, gateway | CCACACCATCGACTTCTTCT |
| MK39.1 | DAYSLEEPER N-term. deletion forward | CCATGGCTGACACTCCGAGAAGG |
| MK40 | DAYSLEEPER N-term. deletion reverse | GGAATTCCTATGCTTCAGAT |
| MK60 | DAYSLEEPER forward primer, gateway | GGGGACAAGTTTGTACAAAAAAGCAGGCTCCATGGAAGTGTACAATGACGATACTG |
| PB01 | DAYSLEEPER forward | ATGGAAGTGTACAATGACGATAC |
| PB02 | DAYSLEEPER reverse | CTATGCTTCAGATTTGATGGTAG |
| SH05 | RHA1 forward primer, gateway | GGGGACAAGTTTGTACAAAAAAGCAGGCTTCATGGCTAGCTCTGGAAACAAGAACA |
| SH06 | RHA1 reverse primers, gateway | GGGGACCACTTTGTACAAGAAAGCTGGGTCAGCACAACACGATGAACTCACTGCC |
